# The Physical and Mechanical Properties of *Arundo donax* (L.) Reeds Affect Their Acoustic Quality

**DOI:** 10.3390/ma18122759

**Published:** 2025-06-12

**Authors:** Jerzy Karczewski, Izabela Potocka, Mario C. De Tullio, Joanna Szymanowska-Pułka

**Affiliations:** 1 Institute of Biology Biotechnology and Environmental Protection, University of Silesia in Katowice, Jagiellońska 28, 40-032 Katowice, Poland; jerzy.karczewski@us.edu.pl (J.K.); izabela.potocka@us.edu.pl (I.P.); 2 Dipartimento di Scienze della Terra e Geoambientali, Università degli Studi di Bari, Via Orabona 4, 70125 Bari, Italy; mario.detullio@uniba.it

**Keywords:** *Arundo donax*, Ashby graph, mechanical properties of plant tissue, sound quality, tissue density

## Abstract

*Arundo donax* (L.) is a perennial monocot (*Poaceae*) native to Asia, which has spread throughout the Mediterranean region. Its hollow stem has been used for millennia to produce reeds, thin strips whose vibration is modulated by musical instruments such as oboes and saxophones. Significant differences in sound quality occur among reeds of different provenances, despite the extreme genetic homogeneity of *A. donax*, mainly due to its clonal mode of reproduction. Reed samples from three different provenances and different sound qualities were analyzed. Samples of dissected internodes of selected brands were examined to determine material density and mechanical properties along the stem radius. These characteristics vary between the brands and change with the sample thickness (along the radius of the stem) according to a power function. Next, Ashby graphs were used to compare the properties of *Arundo* reed samples with those of other natural materials. Using Ashby graphs potentially provides indications for producing musical reeds of the desired sound quality.

## 1. Introduction

*Arundo donax* L., commonly known as a giant reed or giant cane, is a perennial grass species originally native to Asia that then spread to different parts of the world, including the Mediterranean region [1]. A hollow stem of *Arundo* consists of a number of internodes about 20 cm in length and 2–3 cm in diameter [2]. In the cross sections of the tube-like internodes, three concentric bands are visible, formed by (I) a thin ring of the hard waxy epidermis and outer cortical cells, (II) a slightly thicker sclerenchymatous ring including small vascular bundles, and (III) a much thicker inner cortex with parenchyma cells and vascular bundles encircled by sclerified fiber sheaths [3]. Vascular bundles are evenly distributed, unlike in bamboo, where their density increases toward the stem periphery [4]. Either the thickness of the outermost ring of cells (I) or the thickness of the stiff ring of sclerenchyma (II) decreases towards the stem apex. The thickness of the rings (I) and (II) taken together makes up about 8% of the thickness of the whole stem ring [2]. Cell-wall area fraction undergoes a gradual transition along the radius of the stem, rapidly increasing from the outer edge of the stem up to a distance of about 100 μm, then decreasing towards the stem axis [5]. The thickness of the sclerenchyma fibers’ walls in ring II increases gradually down the length of the culm [6].

The plant cell wall is a composite whose major components are hemicellulose and lignin reinforced by oriented cellulose fibrils. The proportion of components depends largely on the type of tissue. In the stem of *Arundo donax*, the highest level of lignification is observed in the sclerenchymatous ring enclosing small vascular bundles (II), and it gradually decreases towards the inner ring (III). The cellulose microfibril orientation remains relatively constant in the cells of ring II but undergoes a sudden change in the parenchymatous tissue of ring III [5]. Due to their composition and structure, these tissues contribute variously to the overall mechanical properties of plant organs. Sclerenchyma is among the stiffest tissues, and its cells, with their high cellulose content, are used as reinforcement in composite materials [7].

It is not possible to characterize the mechanical properties of *Arundo* culm at the macro scale (whole stem) [8] using standard engineering methods because of differences in tissue structure. However, analysis at the meso scale (tissue) shows the effect of anatomical structure on mechanical properties and allows for the determination of the effective Young’s modulus, which is a combination of tissue moduli and tissue fractions [9].

Both the geometrical changeability of the stem and differences in the structure of tissues affect the distribution of mechanical properties within the culm. Tensile tests showed large differences in stiffness of sclerenchymatous and parenchymatous tissues (about 11 GPa and 1 GPa, respectively) and a direct relation between the distance from the outer edge and the Young’s modulus *E* [5]. Also, Young’s modulus decreases towards the apical portion of the stem. In the outer ring comprising bands (I) and (II), Young’s modulus in the longitudinal direction is ten times higher than in the tangential direction. This ratio is even more prominent in band (III) due to this region’s high degree of lignification [2].

For centuries, *Arundo donax* internodes, or their prepared fragments in the form of thin strips, have been used for producing reeds for woodwind instruments such as oboes, bassoons, clarinets, and, more recently, saxophones. While playing, a musician blows air into the instrument, causing vibrations of the reed. In general, the sound quality of a woodwind instrument largely depends on the quality of the reed, but a distinction should be made between single-reed (clarinets and saxophones) and double-reed (oboe and bassoon) instruments (see Supplementary Data, Figure S1). In the latter, the strip is shaped on the sides, then it is folded on itself lengthwise and bound to a staple using a waxed thread. Next, both sides of the reed are scraped, and the tip is cut, forming the opening for air blowing. Most studies conducted to identify a relationship between woodwind sound quality and reed anatomy have considered clarinet reeds [3,10,11]. Oboe reeds have been much less studied [6], although, due to the absence of a mouthpiece like that of clarinets and saxophones, double-reed instruments are likely to be even more dependent on reed quality than single-reed instruments. Each oboe player develops his/her own reedmaking style [12] and usually purchases *Arundo* strips of a given brand whose production meets the musician’s requirements, not only in terms of sound but also with regard to many more parameters, including the responsiveness of the reed (attacks) and its durability (reeds undergo an inevitable decay when played regularly). The material selection for reed production is still based on a somewhat subjective qualitative assessment of the reed-makers, who take into consideration the color of the inside of the cane and of the epidermis, straightness, the length of the internode, and the hardness of the sample tested by knocking two pieces together or by pressing a fingernail into it [6]. Lawton et al. [6] and Kolesik et al. [3] have presented quantitative bases for selecting good-quality reeds made from *Arundo* internode. Lawton et al. [6] indicated that the quality of *Arundo* reeds for oboe is mainly determined by the thickness of the fiber band (II); namely, good reeds come from internodes with the thickest fiber bands, and poor reeds are made from internodes with this band less pronounced. Interestingly, this anatomical feature appeared significantly variable (35–100% of the area occupied by the thickest band measured in a sample cross-section). A study by Kolesik et al. [3] revealed that vascular bundles with large, continuous fiber rings and a low proportion of phloem and xylem tissue within the vascular bundles were responsible for the good musical quality of reeds. It was also reported by both research groups [3,6] that reeds, which, according to the musicians who use them in their instruments, generate sound of high or low quality, and originate from plants in various sites, showed differences in their anatomy.

As in the case of structural engineering materials, the composition and structure of plant cell walls, specifically their thickness and distribution, determine measurable quantitative tissue characteristics, such as density *ρ*, stiffness, and Young’s modulus *E* [13]. For cellular materials, the density of the material as a whole (overall density *ρ*) and the density of the cell walls (solid density *ρ_s_*) are determined. The ratio of these quantities, relative density *ρ*/*ρ_s_*, corresponds to the cell-wall fraction [4]. The Young’s modulus of the tissue, and consequently the tissue stiffness, increases with its overall density [14], which is correlated with the cell-wall thickness [15]. In cells of the tissues that provide the main structural support to a plant, such as sclerified fibers or xylem, the thickness of cell walls increases with the age of the cell due to secondary growth [16], and this is why older tissues are usually stiffer and denser than younger ones.

The properties of materials and their mutual relations are presented in the compact form of the material property (Ashby) charts [13,17,18]. The charts show two properties of materials, for example, density *ρ* and Young’s modulus *E*. Individual materials are represented by bubbles (ellipses) whose sizes refer to the range of the measured value of the property [13]. The Ashby charts are used to analyze the mechanical properties of structures and allow for studying the impact of the reinforcement volume fraction on mechanical properties [19]. By using a logarithmic scale, Ashby charts offer an overview of the wide range of materials and serve as a tool for the optimized selection of materials for design construction elements [14]. The selection of a material for a specific purpose is based on material indices, whose value depends on *E* and *ρ*. For example, a good-quality acoustic material is characterized by low impedance *z* = (*Eρ*)^1/2^ and high radiation coefficient *R* = (*E*/*ρ*^3^)^1/2^ [18,20,21].

The few papers on the mechanical properties of *Arundo* internodes report their variability along both the cross-sectional radius and the stem axis [2,5,22,23]. Additionally, a few works describe their anatomy and the relationship between anatomy and musical performance [3,6]. In this paper, the anatomical, physical, and mechanical features of the reeds of three *Arundo* brands from various locations are compared. Our objective was to investigate whether and how the radially variable structure of the internodal tissues affected these features. To verify this hypothesis, a simple method to measure the mechanical and physical properties of samples of different thicknesses was used. Eventually, to present the relationship between these properties and compare them with other natural materials, Ashby charts were applied. Using Ashby charts, we also aimed to develop a simple method for selecting brands with the desired acoustic properties, characterized by low impedance *z* and high radiation coefficient *R*.

## 2. Materials and Methods

**Figure 1:**
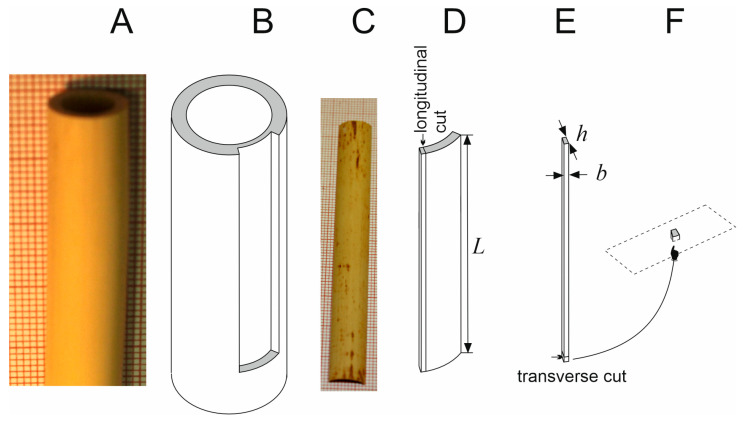
A fragment of the *Arundo* internode prepared for making a reed (**A**) and its schematic representation (**B**) showing a half-moon curved piece for the reed production cut off of the internode; a commercially available curved piece (**C**) for reed construction. Schematic representation showing longitudinal cut (**D**) by which a long, thin sample (**E**) was obtained for density measurement and mechanical tests. Transverse cutting (**E**) was used to obtain a small sample for microscopic analysis (**F**); *h*, *b*, *L*—radial (thickness), circumferential (width), and longitudinal (length) dimensions of the sample.

The samples for the reed construction are prepared such that, from a fragment of the dried internode (Figure 1A), a half-moon curved piece is cut off whose internal cortex band is partly removed (Figure 1B), and then the inside of the specimen is polished. Commercially available dried samples (Figure 1C) to be further processed by musicians to form a reed of a specific shape, which is eventually fixed within the instrument, were used. Three different commercial brands of *Arundo donax* were examined: G, P, and M, originating from southern France.

Thin longitudinal strips (N_G_ = 9, N_P_ = 8, and N_M_ = 4 strips from G, P, and M brands, respectively) were cut from the large fragments (Figure 1D) and used for physical measurements, mechanical tests, and then for microscopic observations. The specimens’ dimensions are given in Table S1.

### 2.1. Microscopic Observations

To obtain cross sections for anatomical analysis, the thin strips were softened in a mixture of 96% ethanol, water, and glycerin (in the volume ratio of 1:1:3) for at least 24 h at room temperature. The strips were washed thoroughly in deionized water and sectioned by hand with a razor blade in the transverse plane (Figure 1E,F). The sections were mounted in water on a microscope slide and observed using a Nikon Eclipse Ni-U microscope (Nikon Instruments Inc., Melville, NY, USA) under bright-field illumination and UV light (UV-2A filter set: excitation filter 330–380, dichroic mirror 400, barrier filter 420). Images were captured with a Nikon Digital Sight DS-Fi1c camera and NIS-Elements F v.4.0 software.

The thickness of the sclerenchyma layer d_s_, referring to the band (II), was determined in the UV fluorescence images using ImageJ 1.53t (open source). Since the thickness of this layer varied along the circumference of the internode, multiple measurements were made on each sample, as shown in Figure S2 (see Supplementary Data). Five images for each brand were used, with about 20 sclerenchyma layer thickness measurements each.

To quantify the anatomical features of the samples observed in their cross-sections, the wall fraction *WF* for individual brands was determined (Figure S3 and description in Supplementary Data) as the ratio of the cell-wall area (*A_s_* solid cross-sectional area) to the total cross-sectional area (*A*) [4].

### 2.2. Sample Density ρ

To determine the sample density, dry thin strips with dimensions of approximately 3 × 0.5 × 92 mm^3^ (width *b* × thickness *h* × length *L*) and mass (*m*) of around 0.2 g (for the exact samples’ dimensions and masses, see Table S1) cut from the large sample fragments (Figure 1D) were used. For individual samples, density (*ρ*) was calculated based on their volume (*V* = *b*·*h*·*L*) and mass (*m*), using the formula *ρ* = *m*/*V* (Δ*ρ* for an individual measurement was taken as the exact differential of *ρ*(*m*, *V*)).

To verify the possible gradual changes of density along sample thickness *h*, we assumed that the relation between *ρ* and *h* is described by the following power function [4]:

(1) $$\rho \left(h\right)=\rho_{0}h^{n},$$

*n* < 0. For each brand, the parameters of this function *ρ*_0_, *n* were determined by data fitting using the MATLAB (R2023a, The MathWorks Inc., Natick, MA, USA) standard procedure (Fit nonlinear regression model-fitnlm).

### 2.3. Young’s Modulus E

**Figure 2:**
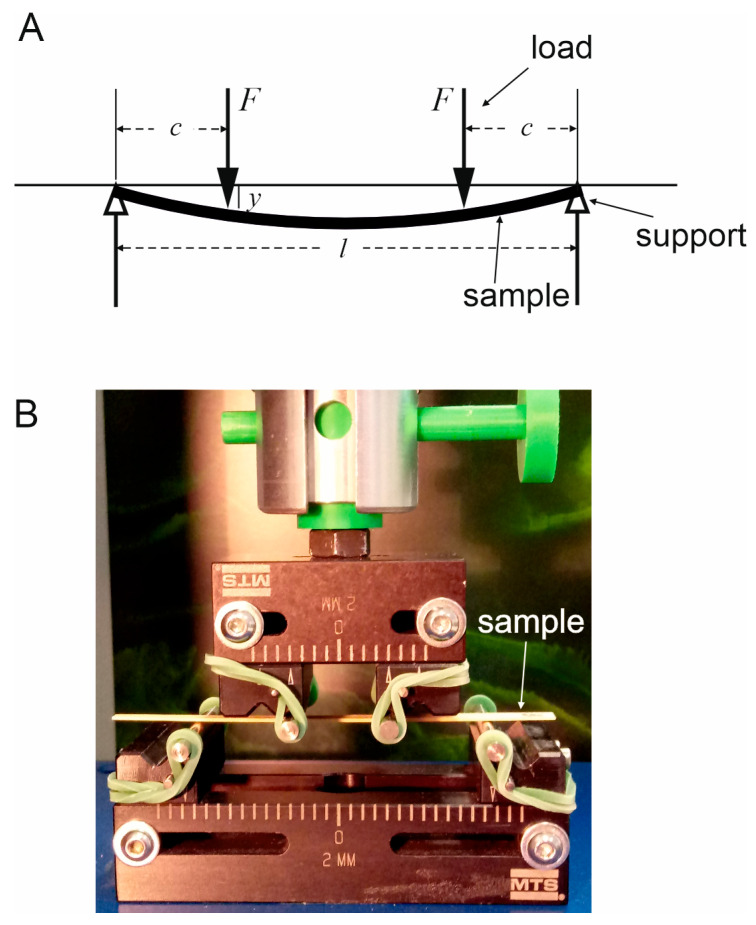
Schematic representation of the 4-point bending test: (**A**), 2*F*—applied load, *l*—spacing between the supporting pins, *c*—distance between the loading pin and supporting pin, and *y*—deflection of the sample measured at the points of load. Close-up of the experiment’s setup (**B**).

The same dry thin strips (Figure 1E) for which *ρ* was determined were used in mechanical tests. To determine Young’s modulus (*E*), we applied the 4-point bending test (Figure 2), the method proposed by Timoshenko [24], using the testing machine SYNERGIE 100 (MTS Systems, Eden Prairie, MN, USA).

In the test, the sample undergoes bending under the known loads *F* (see Figure 2A). The experiment’s setup is shown in Figure S4A (Supplementary Data), and its close-up in Figure 2B. The samples and their orientation in relation to the stem axis are presented in Figures S4B and S4C, respectively. The technical specifications of the equipment used and the experimental conditions are described in the Supplementary Data below Figure S4.

The deflection *y* (a bend of the sample at the points of load) is measured, and Young’s modulus *E* is calculated according to the formula

(2) $$E=\frac{12Fc^{2}}{byh^{3}}\left(\frac{l}{2}-\frac{2}{3}c\right),$$

where *F* = 0.2 N, *c* = 8 mm is the distance between points of load and supports, *l* = 28 mm is the distance between supports, and *b* and *h* are the sample width and thickness, respectively. Deflection *y* was measured 5 to 7 times for each specimen, with a constant loading speed of 1 mm/min. Means ± SD of the deflection *y* for individual samples are shown in Table S1.

To verify the possible gradual changes of Young’s modulus along sample thickness *h*, we assumed that the relation between *E* and *h* would be best described by the following power function [4]:

(3) $$E\left(h\right)=E_{0}h^{n},$$

*n* < 0. For each brand, the parameters of this function *E*_0_, *n* were determined using the MATLAB (R2023a, The MathWorks Inc.) standard procedure (Fit nonlinear regression model—fitnlm).

### 2.4. Statistical Analysis of the Measured Traits

The ANOVA test (significance level *p* = 0.05) and the post hoc least significant differences (LSD) test were used to detect significant differences between the means of the sclerenchyma layer thickness—measured from the UV light images, the means of cell-wall fraction (Supplementary Data, Figures S2 and S3), the means of the measured sample densities, and the means of Young’s moduli, determined by the mechanical test for the three brands of *Arundo*.

### 2.5. Material Property Chart and Acoustical Parameters

Comparing the properties of the *Arundo* samples G, P, and M required the setting of a single objective value for *E* and *ρ* [17]. Since both quantities are assumed functions of *h* (Equations (1) and (3)), the average value of density *ρ_av_* and Young’s modulus *E_av_* was calculated for each brand as a mean of the functions *ρ*(*h*) and *E*(*h*). Based on the mean value theorem [25], the average values of *ρ*(*h*) and *E*(*h*) are given as the integrals of the fitness function over the interval (*h_min_*, *h_max_*)

(4) $$x_{av}=\frac{1}{h_{max}-h_{min}}\int_{h_{min}}^{h_{max}}x\left(h\right)dh,$$

where *x* represents either *ρ* or *E*, and *h_min_* and *h_max_* are the values of the minimal and maximal thickness of the samples of particular brands (see Table S1). For each brand, Δ*ρ_av_* and Δ*E_av_* were calculated as the absolute value of the maximal difference between the experimental (measured *ρ* and *E*) and the model values of *ρ*(*h*) and *E*(*h*), respectively, for each *h*.

To compare the properties of the *Arundo* samples with those of other natural materials, a material property chart [17,26] was constructed using the CES Selector 2015 (Granta Design Limited, Cambridge, UK). In the chart, Young’s modulus *E_av_* is plotted versus material density *ρ_av_* on a logarithmic scale. Various materials (including *Arundo* reed samples G, P, and M) are represented by ellipses whose semi-minor and semi-major axes refer to the range of measured values of Young’s modulus *ρE_av_* and the density of the material Δ*ρ_av_* [13,26]. Young’s modulus (parallel and transverse to the fiber) and the density of wet unprepared *Arundo* internodes are also shown, based on the data from [2,27].

In the log–log plot, Young’s modulus and density dependence on the sample thickness are shown based on experimental data and the regression model log*E*(log*ρ*), using the standard least squares method. Moreover, the selection lines are marked to indicate the material with the desired acoustic properties (minimizing impedance *z* and maximizing radiation *R*). Materials with low *z* are positioned below the line of slope −1, and those with high *R* are placed above the line of slope 3 [18].

Images and plots were edited using CorelDRAW 2020 (Corel Corp., Ottawa, ON, Canada) software.

## 3. Results

### 3.1. Anatomy

**Figure 3:**
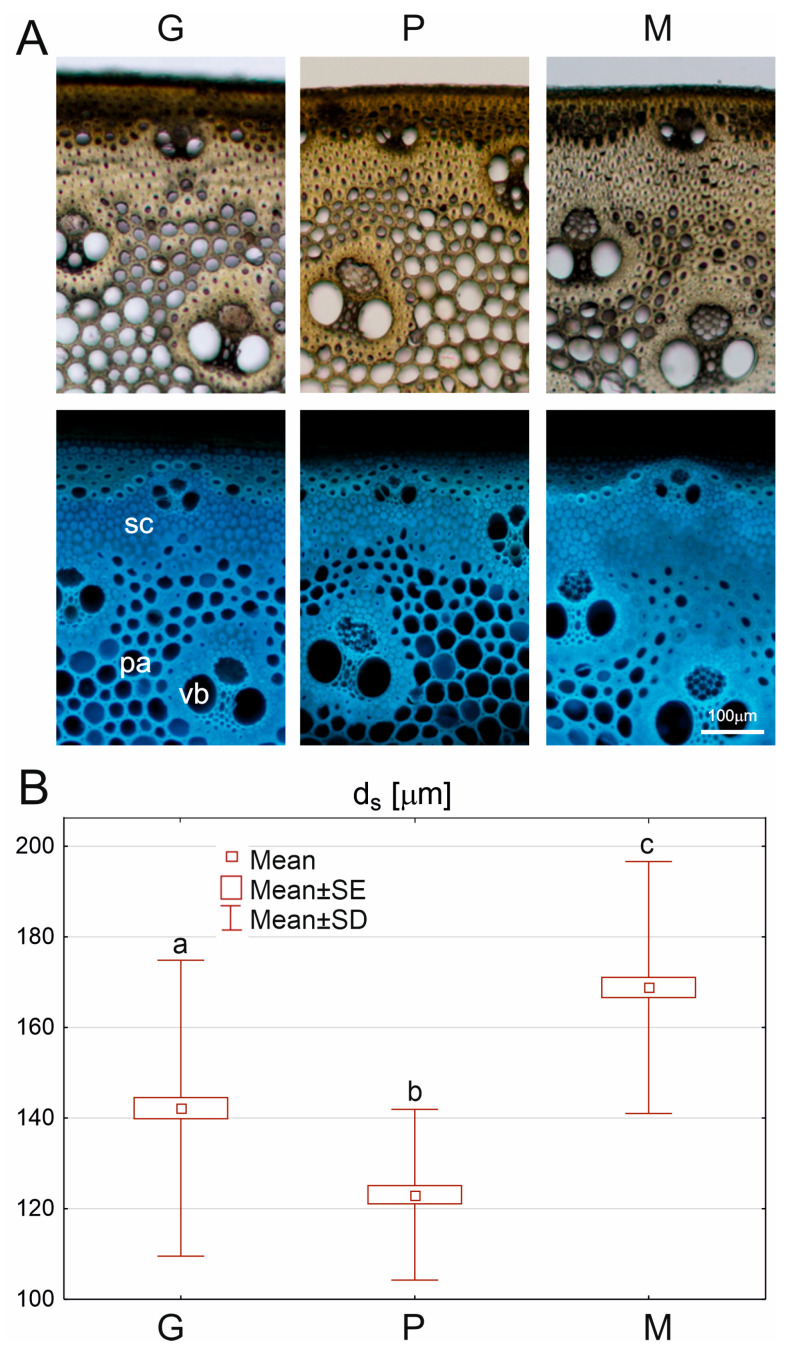
Cross sections through the stem of the three *Arundo* brands (**A**) viewed with bright-field (upper panel) and fluorescence microscopy (lower panel); sc—sclerenchyma, pa—parenchyma, vb—vascular bundles. The measured sclerenchyma layer (d_s_) of the three *Arundo* brands (**B**). Box and whiskers plots represent means (small squares), standard error (SE box), and standard deviation (SD whiskers). The lowercase letters (a, b, and c) above the plots indicate statistically significant differences between the means (comparison between brands using ANOVA and post hoc LSD test, *p* < 0.05).

In Figure 3A, photographs of cross-sections of the internode fragments are shown. In all brands, changes in cell-wall thickness along the radius of the stem are visible. The thickest walls are found in the sclerenchyma fiber band (II) and the sclerenchyma cells around the vascular bundles, while parenchyma cells have thinner walls.

Minor damage/cracks were observed on some sections of the P samples, resulting from preparing the fragile material. Observations under UV light (Figure 3A, lower panel) reveal differences in the thickness of the three brands’ parenchyma cell walls. The thickest parenchyma cell walls are found in the M samples and the thinnest in the P samples. Another characteristic that distinguishes the M samples is that the boundary between the sclerenchymatous ring and parenchyma is less distinct than in other brands, and the layer of thick-walled sclerenchyma cells is the thickest. This last observation is confirmed by quantitative analysis of the thickness of the sclerenchyma band (d_s_) in all brands, which indicates that the differences between the brands are statistically significant (Figure 3B).

A comparison of the mean cell-wall fraction WF for the brands showed that, concerning this trait, the G and P samples are not significantly different, while the WF for M differs from the other two brands (Figure S5). Moreover, the WF shows a gradual transition along the radius, described by a power function that differs for each brand (Figure S6).

### 3.2. Physical and Mechanical Properties

In Table 1, mean densities *ρ*, calculated from the dimensions and mass of the thin strips cut from samples (see Figure 1E), and their Young’s moduli *E*, obtained from Equation (2), are presented. The M samples have the highest and outlying density, while the densities of the two other brands are comparable and not significantly different. The mean Young’s moduli of all three brands differ significantly, with the largest *E* observed in M, the second highest in G, and the smallest in P samples.

**Figure 4:**
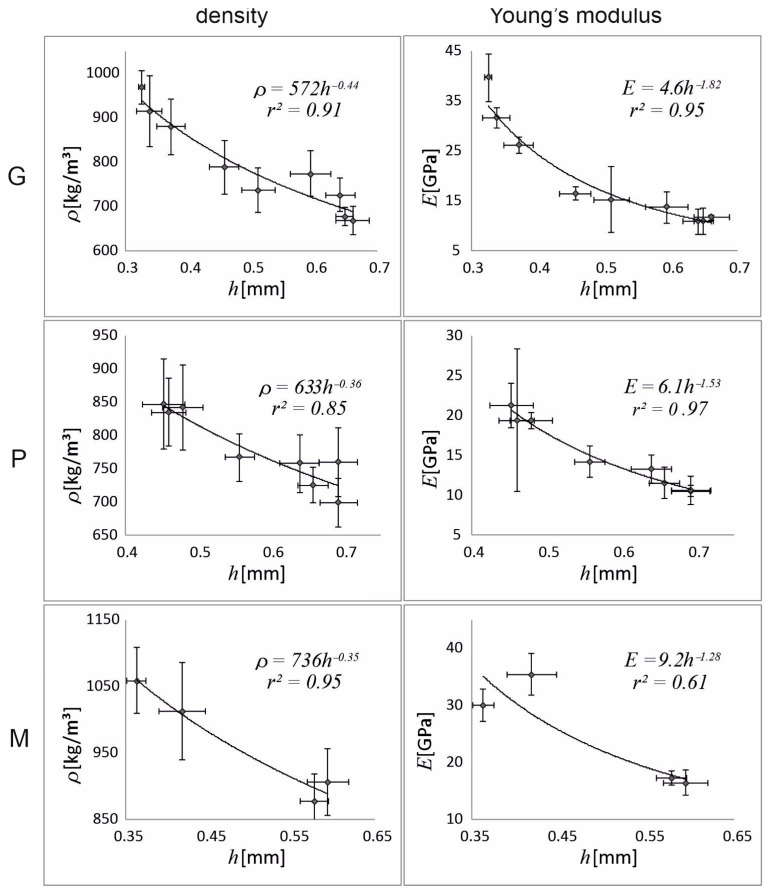
Density ± Δ*ρ* = exact differential of the function *ρ*(*m*, *V*), and Young’s modulus ± SD versus thickness of the sample *h* ± SD for each brand, experiment (dots), and model (solid curve). At the upper-right corner of each plot, the best fitting function *ρ*(*h*), *ρ*(*h*), and coefficient of determination *r*^2^ are given.

Figure 4 plots the experimental data and the model relationship *ρ*(*h*) and *E*(*h*) over the range of measurements for each brand. The equations of the model functions (Equations (1) and (3)) and the coefficient of determination *r*^2^ are given in the upper-right corner. The parameters of the model functions are also presented in Table 2. The fit of the model to the measurement data is generally high (*r*^2^ > 0.8). For *ρ*(*h*), the strongest fit occurs for brand M and the weakest for P, while for *E*(*h*), the strongest fit is for P (*r*^2^ = 0.97) and the weakest for M (*r*^2^ = 0.61).

Based on the mean value theorem (Equation (3) in [25]), the integrals of the fitness functions (Equation (4)) over the interval (*h_min_*, *h_max_*, Table 3) were determined as follows:

(5) $$x_{av}=\frac{x_{0}}{n+1}\frac{h_{max}^{n+1}-h_{min}^{n+1}}{h_{max}-h_{min}},$$

where *x* represents *ρ* or *E*. Next, the average values of *ρ_av_* and *E_av_* were calculated (Table 3). Of the three brands, the M samples show the largest values of both *ρ* and *E*. The average densities of the G and P samples are comparable, while the Young’s moduli appear different; namely, the P samples attain the lowest value of *E_av_*.

### 3.3. Reed Samples’ Position on the Material Property Chart

**Figure 5:**
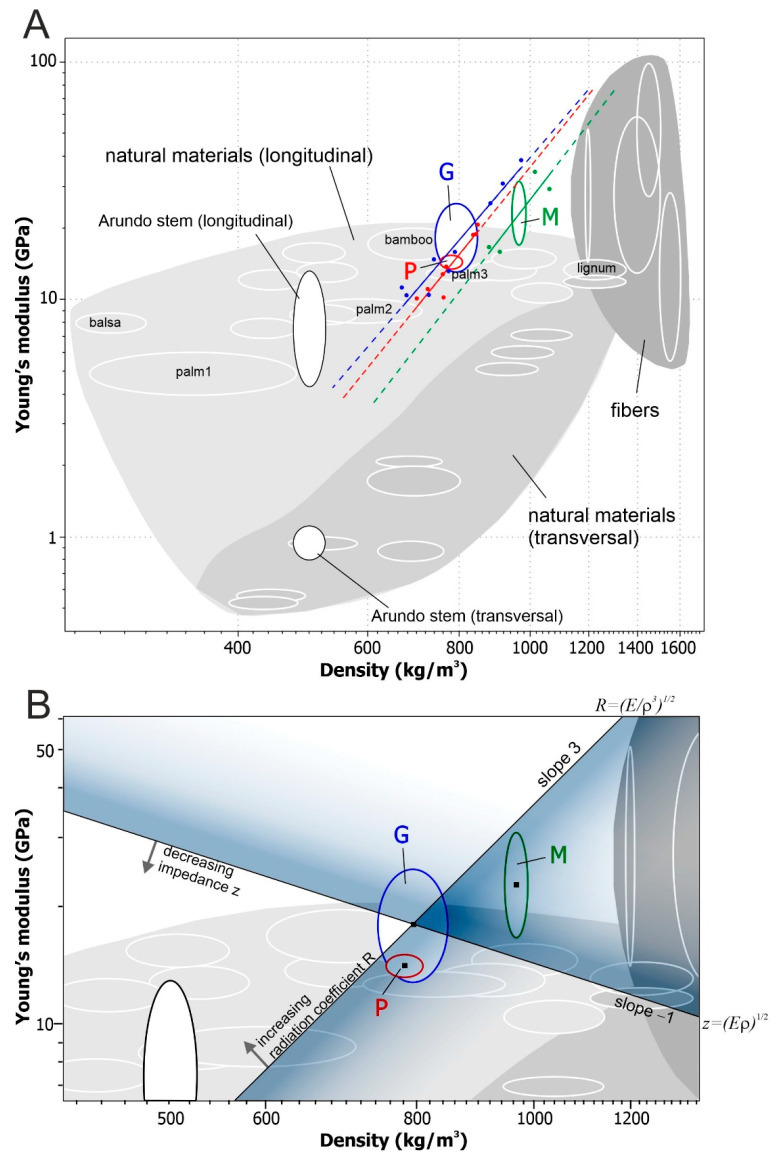
(**A**) Ashby chart plotting Young’s modulus *E* against density *ρ* for natural materials (*E* measured in the longitudinal and transverse direction to the wood fibers). *Arundo* stem in a longitudinal and transverse direction (data from [2,27]), chosen tree and grass species (CES Selector 2015), and reed production samples from the three *Arundo* brands are indicated. The dots represent experimental values of *E* and *ρ* for different sample thicknesses (blue—G, red—P, green—M); regression lines (solid) and prediction lines (dashed fragments) are shown. Palm1, palm2, and palm3 refer to the palm tree stem’s central, inner, and outer tissues, respectively ([4], CES Selector 2015). (**B**) Enlarged fragment of Figure 5A with marked selection lines indicating materials with the best acoustic properties (unshaded area): low impedance—*z* (under the line with slope −1) and high radiation coefficient—*R* (above the line with slope 3).

The Young’s modulus versus density chart in Figure 5 includes ellipses (bubbles) for the three *Arundo* brands (Table 3). In the same way, the Young’s modulus and the density of fresh, wet fragments of *Arundo* internodes are shown (according to [2,27]), as well as of other natural materials (based on the CES Selector database). In the chart, the *Arundo* reed samples are positioned within or near the area of natural materials.

The blue, red, and green dots in Figure 5A indicate experimental values of *E* and *ρ*, determined for samples of different thicknesses. The straight lines show the variation of *E* and *ρ* from the outer edge of the sample (along *h*) for the three brands (different colors). As the thickness of the sample decreases, both *E* and *ρ* increase. The solid lines come from the regression model applied to the experimental data (dots), and the dashed lines, extensions of the regression lines, are extrapolations beyond the original observations.

In Figure 5B, a fragment of the chart from Figure 5A is shown to enable the comparison of the acoustical properties of the samples of the three brands. The selection line of slope −1 represents the impedance *z* = (*Eρ*)^1/2^, while the selection line of slope 3 represents the radiation coefficient *R* = (*E*/*ρ*^3^)^1/2^. Reed samples with high *R* lie above the line of slope 3. Those with low *z* lie below the line of slope −1. Based on this selection, the G brand has the most desirable acoustic properties (minimizing *z* and maximizing *R*) of the three *Arundo donax* brands tested.

## 4. Discussion

A method has been proposed for the simple selection of *Arundo* samples for good sound-quality reeds. The method is quantitative because it allows for the selection of materials with desired acoustic characteristics based on measurable parameters of the samples, that is, Young’s modulus and physical density. Both parameters vary gradually with the *Arundo* stem sample thickness according to a power function.

### 4.1. Reed Samples

In our study, we focused on samples from prepared fragments of internodes (Figure 1C) used by musicians to make reeds. The preparation of these fragments involves gouging, that is, removing the inner parenchyma layers, and requires drying of the material. The thickness of such crafted samples depends on the precision of their shaping by gouging to the desired depth. Thus, the samples of different brands have different thicknesses, which, as shown in this work, affects their different density and Young’s modulus and, consequently, might affect sound quality. Samples of *Arundo* reeds were also used in studies aimed at verifying the hypothesis that their anatomical features were related to the quality of musical performance [3,6]. Other anatomical and mechanical studies usually concern unprepared parts of the internode. Their results indicate similar gradual changes in physical and mechanical properties [2,28].

Whether samples of different brands came from internodes at the same stage of development, we do not know. However, we estimated the radii of the internodes based on the curvature of the outer surface of the samples. They turned out to be similar for all samples, allowing us to assume that the internodes selected for the production of reeds have similar geometric features regardless of the brand.

The structure of samples of *Arundo* stems generally changes along the radius of the cross-sectional area (Figure 3 and [2,3]), which means that they are a structural material. The epidermis and outer cortex enclose sclerenchyma fibers with small vascular bundles and underlying parenchymatous cells with thick walls. The cells of the inner parenchyma layers (located closer to the stem axis) have thinner lignified walls and are larger. In our study, the thickness of the sclerenchyma fiber band appeared brand-dependent (Figure 3). It should also be noted that the thickness of all our samples (Table 3) reaches parenchyma.

Cell-wall area fraction (WF), which is a quantitative measure of differences in structure among the three *Arundo* brands, changes gradually with the distance from the epidermal layer (Figure S6). To describe these changes, a power function model was used, as in the case of density and Young’s modulus. The model fits the experimental data quite well for the G and M samples (*r*^2^ equals about 0.9). The accuracy is low for the P samples (*r*^2^ ≅ 0.5, Figure S6) because, in especially brittle samples of this brand, occasional cracks occurred in the parenchyma cells.

### 4.2. Density and Young’s Modulus Gradient

Mechanical tests have shown that Young’s modulus increases along the radius of the cross-section through the *Arundo* stem from the inner regions towards the peripheries (Figure 4). In the study by Rüggeberg and co-authors [5], Young’s modulus was determined by tensile testing of thin strips of *Arundo* stems cut off with a microtome. This allowed the authors to obtain *E* measurements in the range of 0.1–0.4 mm from the stem’s surface, which was impossible to get in our study due to the use of initially prepared reed samples, the thinnest of which was just over 0.3 mm (Table 3). However, the nature of the change in *E* along the radius of the internode cross-section in the cited work [5] and in our studies was similar within the corresponding range.

The density of dry samples depends on the proportions of their components, mainly cellulose, hemicellulose, and lignin, which are contained in the cell walls. In *Arundo*, the level of cell-wall lignification increases in the direction from the stem axis toward the surface, with the highest occurring in the cells surrounding the vascular bundles and in the sclerenchyma layer [5]. Therefore, the simple relationship between relative density and cell-wall fraction [4] does not apply to reed samples and should be replaced with one that considers the property gradient of this material, meaning a direct dependence of *WF* and *ρ* on the sample thickness, namely *ρ*(*h*)/*ρ_s_*(*h*)~*WF*(*h*).

However, the cell wall’s variable composition and the cellulose microfibrils’ different orientations [5] do not affect the scatter of the density measurements of the samples (small values of the *ρ* coefficient of variation for all brands). At the same time, they may be the reason for the larger scatter of our Young’s modulus measurements, whose CVs for all brands are quite large (Table 1).

### 4.3. From Measurements to Model

To determine Young’s modulus, we applied a standard engineering method, effective for homogeneous materials and specimens with regular shapes. The mechanical properties of structural materials can be determined more accurately using the method described by Greco and coauthors [29] for bamboo stem samples, which involves comparing deflections when loaded in opposite directions. To check for possible differences, we used this method. Yet, for the thin and narrow *Arundo* specimens we tested, the differences in Young’s modulus for the two opposite applications of the bending force appeared minor and not statistically significant.

The method applied in our study allowed us to determine the gradual changes of the mechanical and physical properties of specimens varying in composition and structure. By measuring *E* and *ρ* for the different specimen thickness *h*, it was possible to fit the model *E*(*h*) and *ρ*(*h*) to the measurement points, that is, to determine its parameters *E*_0_, *ρ*_0_, and *n*, which can be regarded as material characteristics. Both the *E*_0_ and *ρ*_0_ parameters (Table 2) refer to the *E*(*h*) and *ρ*(*h*) values for *h* = 1 mm, which remain outside the model range (Table 3). These parameters (*E*_0_ and *ρ*_0_), specific to each brand, determine the set of values that can be achieved by reed shaping. The parameters *n* reflect the rate of change of *E*(*h*) and *ρ*(*h*) with changing *h*. In practice, *n* indicates with what precision the musician should shape the material to obtain a reed of optimal quality (a combination of *ρ* and *E*).

Other material characteristic values for the *Arundo* reed samples are *E_av_*, and *ρ_av_* (Table 3), which are the average *E*(*h*) and *ρ*(*h*) for a selected range of sample thicknesses, calculated similarly to flexural stiffness for a selected range of stem radii in palm and bamboo (see [4]). *E_av_* and *ρ_av_* indicate what values Young’s modulus and density can reach by changing *h_min_* and *h_max_*, that is, how the best *E* and *ρ* can be obtained by modifying the sample thickness. On the other hand, the mean (experimental) values of *E* and *ρ* (Table 1) correspond to the values that a musician most often obtains when preparing reeds from the selected brand. In our study, the values of mean and average *E* and *ρ* are almost equal (compare Table 1 and Table 3), because, as the model limits *h_min_* and *h_max_*, those obtained by shaping were chosen. This indicates that the model (power function) was properly specified. A similar model was already used by [4] when describing the radial modulus gradient of palm and bamboo stems.

Isnard et al. [30] noted that, for a structural material such as plant tissue, the term ‘Young’s modulus’ to describe its stiffness is inaccurate and should be replaced with the term ‘structural Young’s modulus’, referring to the structure’s stiffness as a whole, including the consideration of its distribution. In our research, it is *E_av_* and *ρ_av_* that can be identified with the structural Young’s modulus and the structural density of the *Arundo* samples.

### 4.4. Properties of Unprepared Arundo Stems Versus Properties of Reed Samples

The density of materials of natural origin varies from 270 kg/m^3^ (balsa) to 1200 kg/m^3^ (lignum). Natural fibers are a separate group of materials, and their density in the range of 1200–1600 kg/m^3^ (see Figure 5A, data from CES Selector 2015) corresponds to the density of lignin and cellulose in various proportions. *Arundo* stem has a density of around 500 kg/m^3^, close to the wood of willow, spruce, ash, pine, or the inner part of the trunk of palm trees, which are medium-density materials [4].

The range of densities of each material, indicated on the Ashby chart by the ellipse width, may result from natural individual differences in structure or different environmental conditions. The density of *Arundo* reed samples, obtained by removing the inner cell layers of the stem, ranges from 670 kg/m^3^ to 1060 kg/m^3^ (in Figure 5A, indicated by the positions of the blue, red and green points, obtained from the experiment) and depends on the thickness of the sample and the brand. This wide range of densities, including those of materials such as ash, birch, oak, rosewood, ebony, and the outer tissues of palm trunks and bamboo stems ([4], CES Selector 2015), is not a scatter in the statistical sense, but the result of shaping samples. Thus, shaping *Arundo* samples by removing internal tissues to obtain reeds increases their density so that it approaches that of fibers.

As the material density increases, its Young’s modulus increases, which is particularly evident in the area of natural materials for which the modulus was measured transversely (Figure 5A, middle gray area). This trend is also visible in palms, for which the modulus was measured longitudinally (Figure 5A, palm1, palm2, palm3). The gradual change in density and Young’s modulus along the radius of the sample is shown by the regression lines *E*(*ρ*), individual for each brand (Figure 5A). For *Arundo*, as in the case of palm, the increase in modulus on the property chart (in log–log scale) is proportional to density. So, on a linear scale, the power function describes the relationship between modulus and density.

The dashed sections of the regression line in Figure 5A are extrapolations of the model. The lower sections refer to the thickest samples, while the upper refer to the thinnest. None of the regression lines pass through the area of unprepared *Arundo* stems or natural fibers. This fact is not surprising given that the variation in *E* and *ρ* has been described here by a relatively simple model.

The coordinates of the centers of the ellipses referring to brands G, P, and M in Figure 5B are determined by *ρ_av_* and *E_av_* (Table 3), whose values are similar to the means obtained from the experiment (Table 1). They correspond to the reed thickness most often obtained by the musician in the shaping process. Following [18], we assumed that a high radiation coefficient *R* is crucial for good sound quality. A straight line with a slope of 3, passing through (*ρ_av_*, *E_av_*) of the G brand, determines the highest radiation, so we consider this brand the best in terms of acoustic properties. If our assumption about the importance of radiation for sound is correct, then low density and high material modulus characterize the best material for reeds. However, it is possible that reeds with low impedance sound better to the musician. In such a case, our choice would be P, because the straight line with the smallest z passes through (*ρ_av_*, *E_av_*) of this brand.

Large Young’s modulus at low material density, or in other words the mechanical efficiency of a sample, depends not only on the properties of the material but also on the sample shape (characterized by macrostructural shape factor), the spatial structure (microstructure factor), and the varying volume fraction of material (property gradient [4]).

A final remark concerns an interesting biological issue deriving from this and other studies. *A. donax* essentially reproduces clonally and thus presents very low biological diversity. This may have an epigenetic basis, which might be worth exploring to increase the quality of the raw materials used for reed production.

## 5. Conclusions

Our study investigates how knowledge of the physical and mechanical parameters may help in evaluating the uniformity of the manufacturing, and, from the point of view of a musician, the choice of the materials that give the best results in terms of sound, attacks, durability, and so on, according to his/her own needs and requirements.

By determining *E* and *ρ* for the samples, an Ashby chart was constructed, which made it possible to reveal a difference in the physical and mechanical properties of natural materials, including fresh, undried *Arundo* stem fragments and prepared *Arundo* samples. The drying of the material and the removal of the inner tissue layers significantly changed the properties, making *Arundo* samples, whose structure is heterogeneous, a good material for reed production. The material indices (performance indices) *z* and *R*, which are combinations of density and modulus, are useful for selecting the best *Arundo* brand for manufacturing reeds.

The variability in cell-wall composition and the relatively small number of measurements do not significantly affect the fit of the model to the measurements, since the coefficients of determination *r*^2^ for both *E*(*h*) and *ρ*(*h*) are high for all brands. This allows us to state that in the range of the thickness of the tested samples (*h_min_*, *h_max_*), the power function is a good interpolation of the real relationship between *E*(*h*) and *ρ*(*h*).

The novelty of our results is the proposed simple method for selecting a material with good acoustic properties based on the known density and mechanical properties of the material. The standard-based 4-point bending test applied to specimens of different thicknesses has yielded good results in determining the Young’s modulus of the structural material. The paper also determines the parameters of a curvilinear model describing changes in cell-wall fraction, density, and Young’s modulus with sample thickness for the reeds of three commercial *Arundo* brands.

## Figures and Tables

**Table 1:** Mean densities *ρ* ± SD and mean Young’s moduli *E* ± SD of the three *Arundo* brands with coefficients of variation CV. Different letters (a, b, and c) indicate statistically significant differences between the means (comparison between brands using ANOVA and post hoc LSD test, *p* < 0.05).

Brand	*ρ* ± SD	CV	*E* ± SD	CV
G	792.5 ± 100.1 a	0.13	19.6 ± 9.8 a	0.50
P	778.9 ± 52.4 a	0.07	15.0 ± 4.1 b	0.27
M	963.3 ± 74.4 b	0.08	24.8 ± 8.2 c	0.33

**Table 2:** Parameters of the model functions *ρ*(*h*) and *E*(*h*) for particular brands.

	*ρ*(*h*) = *ρ*_0_*h^n^*		*E*(*h*) = *E*_0_*h^n^*	
Brand	*ρ*_0_	*n*	*E*_0_	*n*
G	572 ± 22	−0.44 ± 0.05	4.6 ± 0.7	−1.82 ± 0.16
P	633 ± 21	−0.36 ± 0.06	6.1 ± 0.4	−1.53 ± 0.10
M	736 ± 28	−0.35 ± 0.05	9.2 ± 4.8	−1.28 ± 0.59

**Table 3:** Minimum *h_min_* and maximum *h_max_* sample thickness ± measurement accuracy = SD, average density *ρ_av_* ± Δ*ρ_av_*, and Young’s modulus *E_av_*± Δ*E_av_* obtained from the model (Equation (5)) for the reed samples of the analyzed brands. Δ*ρ*_a,_ and Δ*E*_av_ are absolute values of the maximal difference between the experimental and model values (within the range of *h_min_* to *h_max_*, see Table S1) of *ρ*(*h*) and *E*(*h*), respectively.

Brand	*h_min_* ± Δ*h_min_* [mm]	*h_max_* ± Δ*h_max_* [mm]	Average Density *ρ_av_* ± Δ*ρ_av_* [kg/m^3^]	Average Young’s Modulus *E_av_* ± Δ*E_av_* [GPa]
G	0.326 ± 0.005	0.661 ± 0.026	792 ± 53	19 ± 6
P	0.453 ± 0.029	0.692 ± 0.026	779 ± 35	15 ± 1
M	0.363 ± 0.012	0.594 ± 0.025	965 ± 21	24 ± 7

## Data Availability

The data presented in this article are not readily available because further research will be conducted using them. Request to access the data should be directed to the corresponding author.

## Supplementary Materials

The following supporting information can be downloaded at https://www.mdpi.com/article/10.3390/ma18122759/s1: Figure S1: Examples of single and double reeds. Figure S2: A method of measuring the thickness of the sclerenchyma layer d_s_; e—epidermis, s—sclerenchyma, p—parenchyma. Figure S3: Cell-wall fraction measurement; UV image of a cross-section through the sample (A) converted to grayscale image (B), converted to binarized image (C). The red grid in (C) shows the regions where the cell-wall fraction was determined. The position of the grid relative to the cell pattern was determined along the *h*-axis (sample thickness). The distance between the grid’s vertical lines is 0.05 mm, and the grid height is 1 mm. Figure S4: (A) The experimental setup. Testing machine with a sample prepared for the test. The screen next to the machine and the inset in the lower-right corner show close-ups of the sample from different angles. (B) Specimens of the brands (G, P, M) in internal, side, and external (stem surface) views. (C) Color-coded directions in reference to the stem axis, also shown on the close-ups in (A) and on the P specimens in (B). Figure S5: The means of *WF* of the three *Arundo* brands. Box and whiskers plots represent means (small squares), standard error (SE box), and standard deviation (SD whiskers). The same lowercase letters (a, b) above the plots indicate statistically insignificant differences between the means (comparison between brands using ANOVA and post hoc LSD test, *p* < 0.05). Figure S6: Cell-wall fraction ± SD versus thickness of the sample *h* ± SD for each brand, experiment (dots), and model (solid curve). At the upper-right corner of each plot, the model function WF(h) and the coefficient of determination *r^2^* are given. Table S1: Mean values ± SD (standard deviation of the mean) of the sample width *b* (circumferential) and thickness *h* (radial), sample length *L* (longitudinal) ± accuracy of the digital calipers, sample mass *m* ± accuracy of the laboratory scales, mean value of sample deflection *y* ± SD, measured in mechanical tests. In bold, the *h_min_* and *h_max_* for each brand are indicated. Description of *WF* measurement method and results.

## Abbreviations

The following abbreviations and symbols are used in this manuscript:

SD	Standard deviation
CV	Coefficient of variation
(I), (II), (III)	Three concentric bands formed by cells of different tissues, visible in cross-section through the *Arundo* internode: (I) an outermost thin ring of the hard waxy epidermis and outer cortical cells, (II) a slightly thicker sclerenchymatous ring including small vascular bundles, and (III) a thick inner cortex with parenchyma cells and vascular bundles encircled by sclerified fiber sheaths
G, P, M	Three different commercial brands of *Arundo donax* used in the experiments
d_s_	The thickness of the sclerenchyma layer, referring to band (II) in the cross-sectional view of the stem
*h*	Specimen thickness measured along the radius of the stem
*b*	Specimen width measured in the circumferential direction
*L*	Specimen length measured in the longitudinal direction
*V*	Specimen volume calculated using the formula *V* = *b*·*h*·*L*
*A*	The total cross-sectional area of the specimen with a porous structure
*A_s_*	The solid cross-sectional area of the specimen with a porous structure
*WF*	Cell-wall fraction calculated as the ratio of *A_s_* to *A*
*m*	Specimen mass
*ρ*	The overall density of the specimen calculated using the formula *ρ* = *m*/*V*
*ρ**_s_*	The density of the cell walls = solid density
*F*	The load applied in the mechanical test
*c*	The distance between the loading pin and the supporting pin in the mechanical test
*l*	The distance between supports in the mechanical test
*y*	Specimen deflection measured in the mechanical test
*E*	Young’s modulus calculated using the formula $E=\frac{12Fc^{2}}{byh^{3}}\left(\frac{l}{2}-\frac{2}{3}c\right)$
N_G_, N_P_, N_M_	Number of specimens of particular *Arundo* brands used in density measurements and mechanical tests
*h_min_*, *h_max_*	Minimal and maximal thickness of the specimens of particular brands
*ρ**_av_*	Average density, mean of the function *ρ*(*h*)
*E_av_*	Average Young’s modulus, mean of the function *E*(*h*)
*z*	The acoustic impedance of the material *z* = (*Eρ*)^1/2^
*R*	Radiation coefficient *R* = (*E*/*ρ*^3^)^1/2^
*p*	Probability in statistical tests
*r*^2^	Coefficient of determination
sc	Sclerenchyma
pa	Parenchyma
vb	Vascular bundle
