# Supplementary material for: The Physical and Mechanical Properties of Arundo donax (L.) Reeds Affect Their Acoustic Quality
Source: Materials (Basel). 2025 Jun 12;18(12):2759. doi: 10.3390/ma18122759 (PMC12195560; doi:10.3390/ma18122759)
Supplement: Supplementary file 1 [file materials-18-02759-s001.zip › materials-3504415-supplementary.pdf]

Supplementary Data

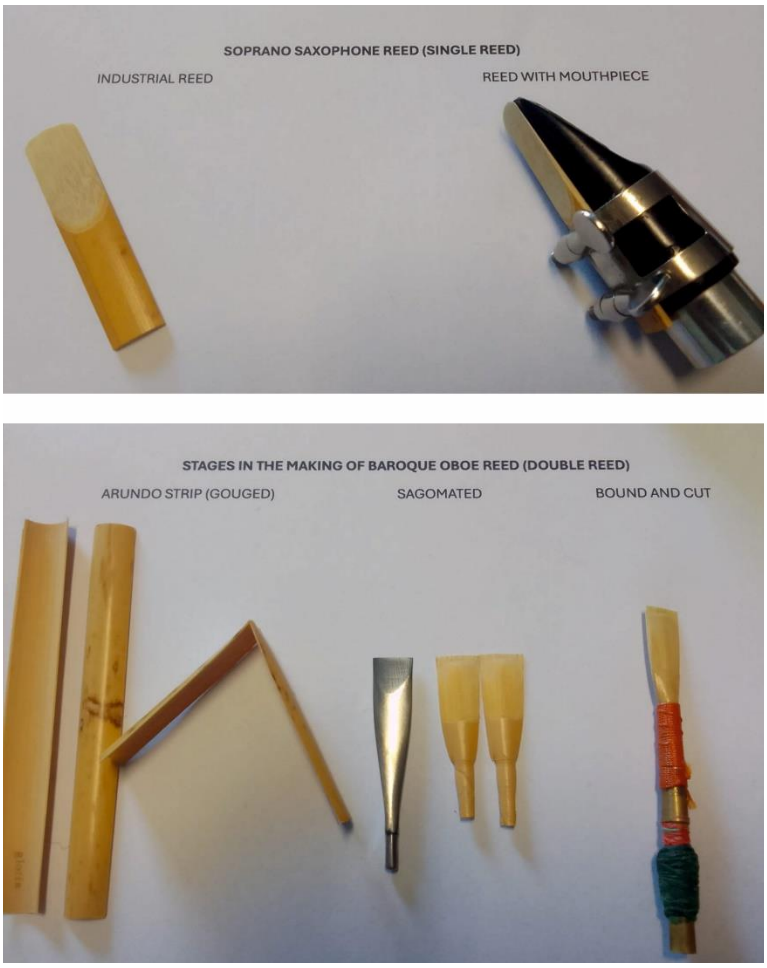


**Figure S1**. Examples of single and double reeds

Specimen dimensions, mass, and deflection

Width ($b$) and thickness ($h$) of the samples (Figure 1E) were measured with the use of a micrometer, 5 measurements of width and 10 measurements of thickness along the sample. Mean values ±SD (standard deviation of the mean) of $b$ and $h$ obtained for each sample were applied for further calculation. Length ($L$) was measured using digital calipers ($\Delta L$ = 0.01 mm), once for each sample. Mass was measured using the precision laboratory scales ($\Delta m$ = 0.0001g).

| brand | specimen No | $b\pm\Delta b$ [mm] | $h\pm\Delta h$ [mm] | $L\pm\Delta L$ [mm] | $m\pm\Delta m$ [g] | $y\pm\Delta y$ [mm] |
|---|---|---|---|---|---|---|
| G | 1 | 2.588±0.019 | **0.661±0.026** | 92.09±0.01 | 0.1052±0.0001 | 0.155±0.008 |
| | 2 | 2.660±0.021 | 0.648±0.013 | 92.15±0.01 | 0.1075±0.0001 | 0.171±0.011 |
| | 3 | 2.932±0.064 | **0.326±0.005** | 91.97±0.01 | 0.0851±0.0001 | 0.337±0.043 |
| | 4 | 2.226±0.031 | 0.511±0.027 | 92.04±0.01 | 0.0772±0.0001 | 0.298±0.031 |
| | 5 | 2.904±0.069 | 0.457±0.024 | 92.11±0.01 | 0.0964±0.0001 | 0.296±0.021 |
| | 6 | 2.710±0.071 | 0.338±0.020 | 92.02±0.01 | 0.0771±0.0001 | 0.406±0.026 |
| | 7 | 3.810±0.038 | 0.594±0.032 | 92.10±0.01 | 0.1613±0.0001 | 0.123±0.006 |
| | 8 | 3.446±0.053 | 0.641±0.023 | 92.08±0.01 | 0.1476±0.0001 | 0.137±0.006 |
| | 9 | 3.056±0.033 | 0.372±0.022 | 92.01±0.01 | 0.0920±0.0001 | 0.326±0.019 |
| P | 1 | 3.798±0.055 | **0.692±0.026** | 92.64±0.01 | 0.1701±0.0001 | 0.102±0.007 |
| | 2 | 3.422±0.033 | 0.557±0.020 | 93.04±0.01 | 0.1360±0.0001 | 0.162±0.019 |
| | 3 | 3.462±0.058 | 0.479±0.028 | 92.97±0.01 | 0.1297±0.0001 | 0.182±0.010 |
| | 4 | 2.852±0.086 | 0.692±0.026 | 93.17±0.01 | 0.1397±0.0001 | 0.138±0.029 |
| | 5 | 3.240±0.016 | 0.657±0.021 | 92.91±0.01 | 0.1434±0.0001 | 0.132±0.030 |
| | 6 | 3.582±0.051 | **0.453±0.029** | 92.84±0.01 | 0.1276±0.0001 | 0.193±0.029 |
| | 7 | 2.932±0.028 | 0.460±0.023 | 92.81±0.01 | 0.1045±0.0001 | 0.243±0.013 |
| | 8 | 3.724±0.054 | 0.639±0.027 | 92.92±0.01 | 0.1676±0.0001 | 0.107±0.017 |
| M | 1 | 3.240±0.012 | 0.418±0.028 | 91.98±0.01 | 0.1261±0.0001 | 0.162±0.015 |
| | 2 | 3.156±0.040 | **0.594±0.025** | 91.93±0.01 | 0.1562±0.0001 | 0.126±0.015 |
| | 3 | 3.172±0.054 | 0.578±0.017 | 91.96±0.01 | 0.1478±0.0001 | 0.129±0.010 |
| | 4 | 2.092±0.028 | **0.363±0.012** | 91.97±0.01 | 0.0739±0.0001 | 0.448±0.041 |

**Table S1**. Mean values ± SD (standard deviation of the mean) of the sample width $b$ (circumferential) and thickness $h$ (radial), sample's length $L$ (longitudinal) ± accuracy of the digital calipers, sample's mass $m$ ± accuracy of the laboratory scales, mean value of sample deflection $y$ ± SD, measured in mechanical tests. In bold, the $h_{min}$ and $h_{max}$ for each brand are indicated.

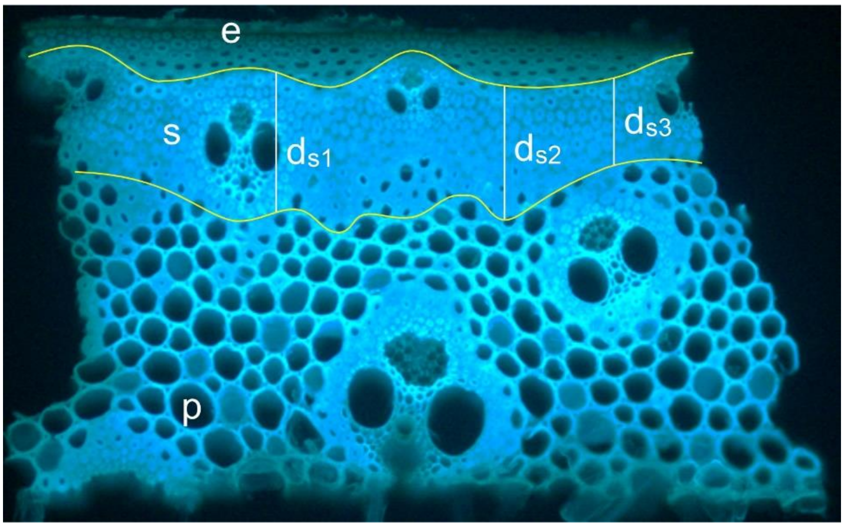


**Figure S2**. A method of measuring the thickness of the sclerenchyma layer $d_s$; e epidermis, s sclerenchyma, p parenchyma.

Testing machine, specimens, and conditions

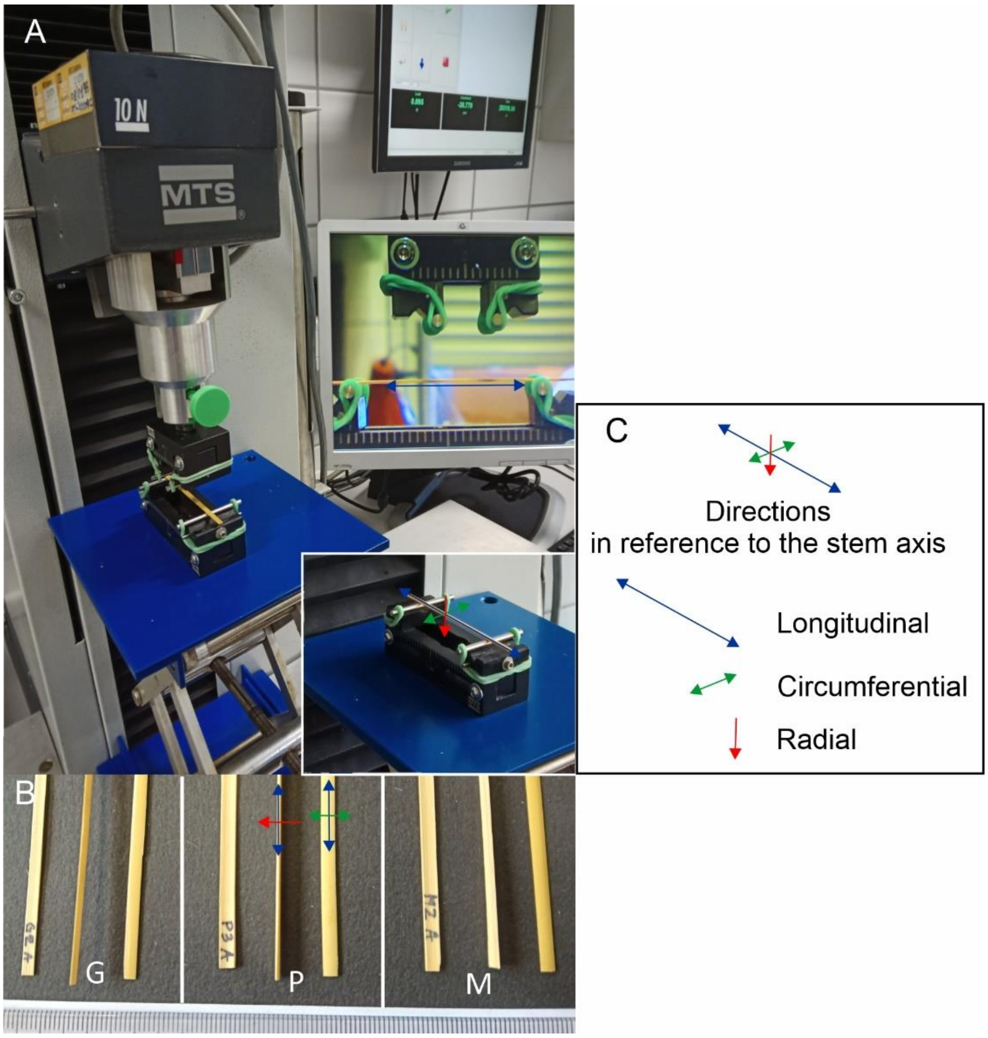


**Figure S4.** (A) The experimental setup. Testing machine with a sample prepared for the test. The screen next to the machine and the inset in the lower right corner show close-ups of the sample from different angles. (B) Specimens of the brands (G, P, M) in internal, side, and external (stem surface) views. (C) Color-coded directions in reference to the stem axis, also shown on the close-ups in (A) and on the P specimens in (B).

Experimental setup specification: Universal (tensile and compression) testing machine MTS SYNERGIE 100 equipped with a load cell of 10N, accuracy load measurement 0.5%, and a resolution of 0.0001 N. Crosshead displacement accuracy 0.1% and a resolution of 0.01 mm

Conditions under which the measurements were carried out: Temperature 20±2 °C, RH=30±5 %

*Cell wall fraction*

In the cross-section through the stem, wall fraction (*WF*) is calculated as the ratio of the cell wall area (solid cross-sectional area, $A_s$) to the total cross-sectional area ($A$), namely $A_s/A = WF$ [4]. Using ImageJ, UV fluorescence images of cross-sections through the internodes (Figure S3A), were converted into grayscale images (Figure S3B). Next, they were binarized (converted into black-and-white images) using the MATLAB (*R2023a,* The MathWorks Inc.) standard procedure *imbinarize*, thus becoming a set of black and white pixels (Figure S3C). As a result, the cell walls were coded white.

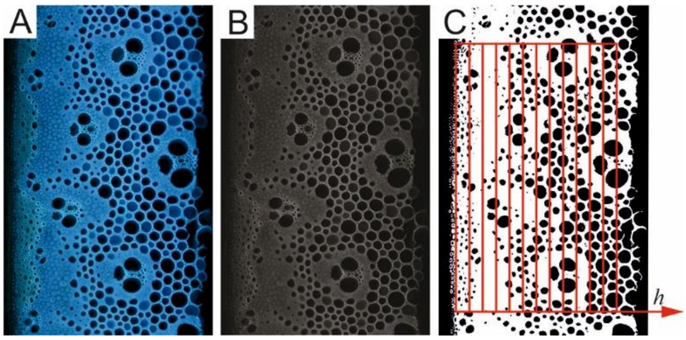


**Figure S3.** Cell wall fraction measurement. UV image of a cross-section through the sample (**A**) converted to grayscale image (**B**), converted to binarized image (**C**). The red grid in (**C**) shows the regions where the cell wall fraction was determined. The position of the grid relative to the cell pattern was determined along the $h$-axis (sample thickness). The distance between the grid's vertical lines is 0.05 mm, and the grid height is 1 mm.

A 1-mm-long grid composed of up to 12 rectangles 0.05 mm wide was superimposed on a cross-section starting from the peripheral zone (Figure S3C). The grid comprised at least ten cells (see [5]) along the radius of the specimen, that is, along the $h$ direction. The position of the rectangle along the radial dimension was measured from the outer edge of the transversely sectioned sample (Figure S3C) to the accuracy of $\Delta h$ = 0.025 mm, that is, half the width of the rectangle. White pixels were counted in a rectangle, and their number $A_s$ divided by the number of all pixels in rectangle $A$ determined the fraction of cell walls (part of the area occupied by cell walls) $WF = A_s/A$ in $h$ position. Throughout the procedure, the same parameters were maintained for the images of the sample sections of all brands. Measurements were repeated on 6 cross-sections for each brand; the final result was $WF$ ± $SD_{max}$, where $WF$ was the mean value of the cell wall fraction in the corresponding rectangular area of the six samples and $SD_{max}$ was the maximum of the six SDs. The cell wall fraction (*WF*) distribution was analyzed versus the sample thickness $h$ every 0.05 mm in the 0.1-0.6 mm range, in which the fraction decreases. The ANOVA test (significance level $p$ = 0.05), followed by the *post hoc* Least Significant Differences (LSD) test were used to detect significant differences between the means of the cell wall fraction for the three brands of *Arundo*.

The relation between $WF$ and $h$ was assumed to be a power function $WF(h) = WF_0 h^n$. For each brand, the parameters of this function $WF_0$, $n$ were determined using the MATLAB (R2023a, The MathWorks Inc.) standard procedure (Fit nonlinear regression model – fitnlm).

The proportion of cell wall area to total cross-sectional area (cell wall fraction) was, on average, about 90% in the outermost tissues (sclerenchyma) and about 50% in the innermost tissues of the internodes. Mean fractions varied for three brands (Figure S4), and $WF$ for M proved to be statistically significantly different from the $WF$ of the other brands.

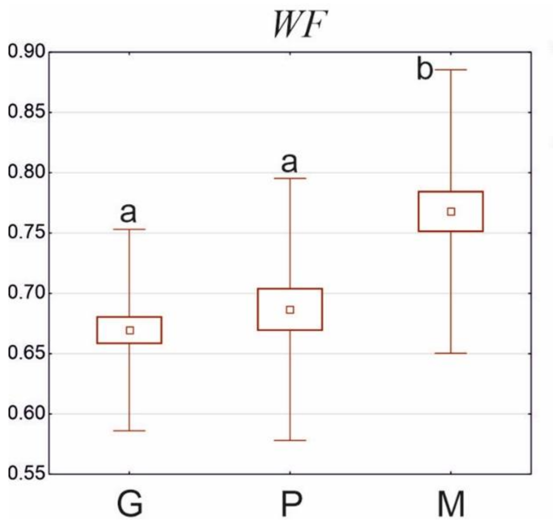


**Figure S5**. The means of $WF$ of the three *Arundo* brands. Box and whiskers plots represent means (small squares), standard error (SE box), and standard deviation (SD whiskers). The same small letters (a, b) above the plots indicate statistically insignificant differences between the means (comparison between brands using ANOVA and *post hoc* LSD test, $p < 0.05$).

Relations between the cell wall fraction and sample thickness for each brand are presented in Figure S5. A power function describes the trend.

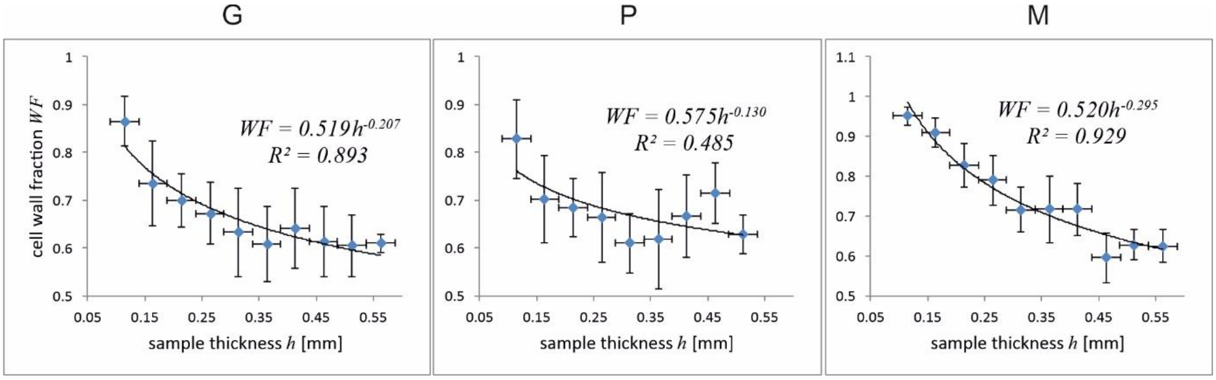


**Figure S6**. Cell wall fraction ± SD versus thickness of the sample $h$ ± SD for each brand, experiment (dots), and model (solid curve). At the upper right corner of each plot, the model function WF(h) and the coefficient of determination $r^2$ are given.
